# Multifocal motor neuropathy secondary to gluten intolerance: a case report

**DOI:** 10.1186/s13256-026-05968-2

**Published:** 2026-03-26

**Authors:** K. Haddouali, K. Simma, S. Bellakhdar, H. El Otmani, B. El Moutawakil, M. A. Rafai

**Affiliations:** 1 Department of Neurology, Ibn Rochd Hospital, Faculty of Medicine and Pharmacy, Hassan II University, Tarik Ibnou Ziad Street, 20250 Casablanca, Morocco; 2 Genetics Laboratory, Faculty of Medicine and Pharmacy, Hassan II University, Tarik Ibnou Ziad Street, 20250 Casablanca, Morocco; 3 Research Laboratory on Diseases of the Nervous System, Neurosensory and Handicap, Faculty of Medicine and Pharmacy, Hassan II University, Tarik Ibnou Ziad Street, 20250 Casablanca, Morocco; 4 Department of Neurology, Ibn Rochd University Hospital of Casablanca, Tarik Ibnou Ziad Street, 20250 Casablanca, Morocco

**Keywords:** Gluten-related disorders, Multifocal motor neuropathy, Gluten-free diet

## Abstract

**Background:**

Peripheral nervous system manifestations of gluten sensitivity usually present as distal symmetric axonal polyneuropathy, small fiber neuropathy, or sensory neuropathy, often accompanied by ataxia or painful symptoms. By contrast, motor neuropathies are extremely rare, with only a few cases of multifocal motor neuropathy reported. Notably, the most recent guidelines of the European Society for the Study of Coeliac Disease do not recognize multifocal motor neuropathy as a classical neurological manifestation of gluten-related disorders. The main differential diagnoses include amyotrophic lateral sclerosis and chronic inflammatory demyelinating polyneuropathy. Currently, strict adherence to a gluten-free diet remains the only therapeutic option. Although clinical improvement is not universal, when observed, it strongly supports the causal role of gluten-related neurotoxicity.

**Case presentation:**

We report the case of a 50-year-old North African man with a history of dermatitis herpetiformis and persistent chronic diarrhea. Over the past 5 years, he developed progressive asymmetric motor weakness. Clinical examination revealed an asymmetric, pure motor, peripheral neurogenic syndrome, confirmed by nerve conduction studies, without evidence of proximal conduction block. Cerebrospinal fluid analysis showed elevated protein levels (0.75 g/L), and serum anti-GD3 IgM antibodies were positive. Brachial plexus magnetic resonance imaging revealed bilateral hypertrophy on T1-weighted sequences, with hyperintensity on short tau inversion recovery sequences but no contrast enhancement. The motor deficit gradually improved after the introduction of a gluten-free diet. The patient also received intravenous immunoglobulin without significant clinical benefit. On the basis of clinical, paraclinical, and follow-up findings, a diagnosis of multifocal motor neuropathy secondary to gluten intolerance was established.

**Conclusion:**

This case highlights the importance of considering gluten intolerance as a potential etiology, particularly given the reversible nature of this neuropathy, which can otherwise be misdiagnosed as a more severe condition such as amyotrophic lateral sclerosis. Further studies are needed to better elucidate the pathophysiology of this rare gluten-related neurological phenotype.

## Background

The spectrum of peripheral nervous system manifestations associated with gluten sensitivity most commonly includes distal symmetric axonal polyneuropathy, small fiber neuropathy, or sensory neuropathy, frequently accompanied by ataxia or painful symptoms [1]. By contrast, motor neuropathies are exceedingly rare, with only a few cases of multifocal motor neuropathy (MMN) reported in literature [2]. Notably, the most recent guidelines of the European Society for the Study of Coeliac Disease (ESsCD) do not recognize MMN as a classical neurological manifestation of gluten-related disorders (GRD), reflecting the very limited number of well-documented cases available. The main differential diagnoses in this context include amyotrophic lateral sclerosis (ALS) and chronic inflammatory demyelinating polyneuropathy (CIDP), both of which may present with overlapping clinical features. The pathophysiology of gluten-related neuropathies, particularly pure motor forms, remains incompletely understood. Several mechanisms have been proposed, including antibody cross-reactivity and metabolic factors. Increasing evidence supports the role of immune-mediated processes, notably antibody cross-reactivity with neural antigens such as transglutaminase 6 (which has been proposed as a key autoantigen in gluten-related neurological disorders [3]. Currently, a strict gluten-free diet (GFD) remains the cornerstone of treatment. Although clinical improvement is not consistently observed, when present, it strongly supports a causal role of gluten-related neurotoxicity. While immunotherapy is frequently used and often effective in classical MMN, its role in gluten-related pure motor neuropathies remains unclear and is not supported by robust evidence. The rarity of this condition continues to pose significant challenges for diagnosis and management, underscoring an important knowledge gap that the present case aims to address.

## Case presentation

**Fig. 1:**
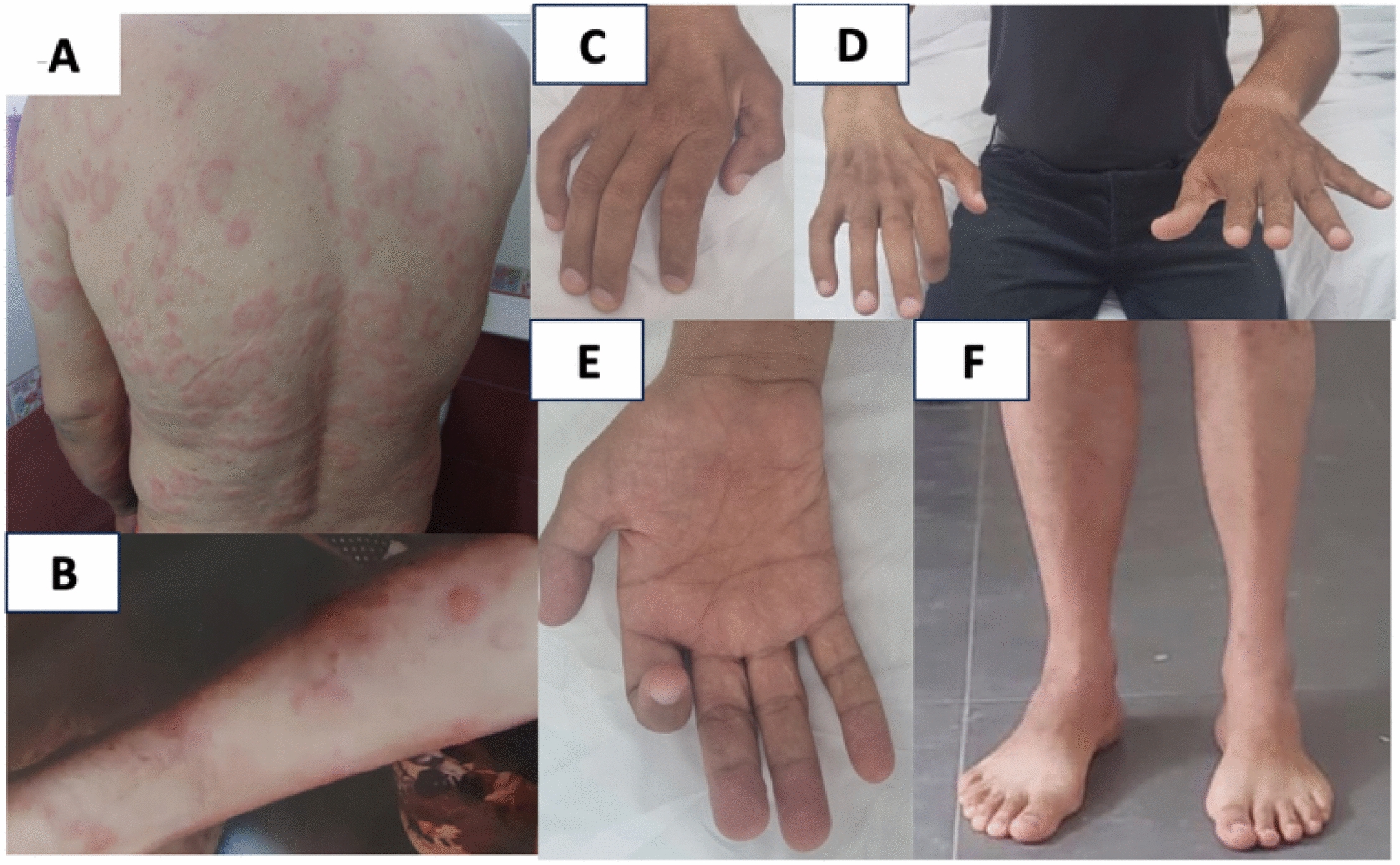
Patient’s clinical features. Diffuse dermatitis herpetiformis involving the trunk (**A**) and limbs (**B**). Asymmetric, multitruncular amyotrophy affecting all four limbs, predominantly involving the intrinsic muscles of the right hand (**C**–**E**) and the left leg (**F**)

We report the case of a 50-year-old North African man with a history of diffuse, corticosteroid-sensitive dermatitis herpetiformis (DH) associated with chronic diarrhea. Over the preceding 5 years, he developed a progressive and disabling motor deficit, initially affecting the right upper limb and extending to the contralateral lower limb within 6 months (Fig. 1A). Given this pure motor presentation, three neurologists initially considered a diagnosis of ALS. As the motor weakness progressed, the patient experienced a relapse of DH. He noticed a clear worsening of neurological and dermatological symptoms during ongoing gluten exposure and therefore voluntarily initiated a strict GFD. This intervention resulted in complete remission of DH and diarrhea, along with marked, though incomplete, improvement in motor function. At 5 months after initiating the GFD, neurological examination at our department revealed a pure motor, areflexic deficit with asymmetric weakness and muscle atrophy involving all four limbs, consistent with a multiple mononeuropathy pattern (Table 1). The patient remained ambulatory, with no sensory symptoms or fasciculations, and a normal cranial nerve examination.

**Table 1:**
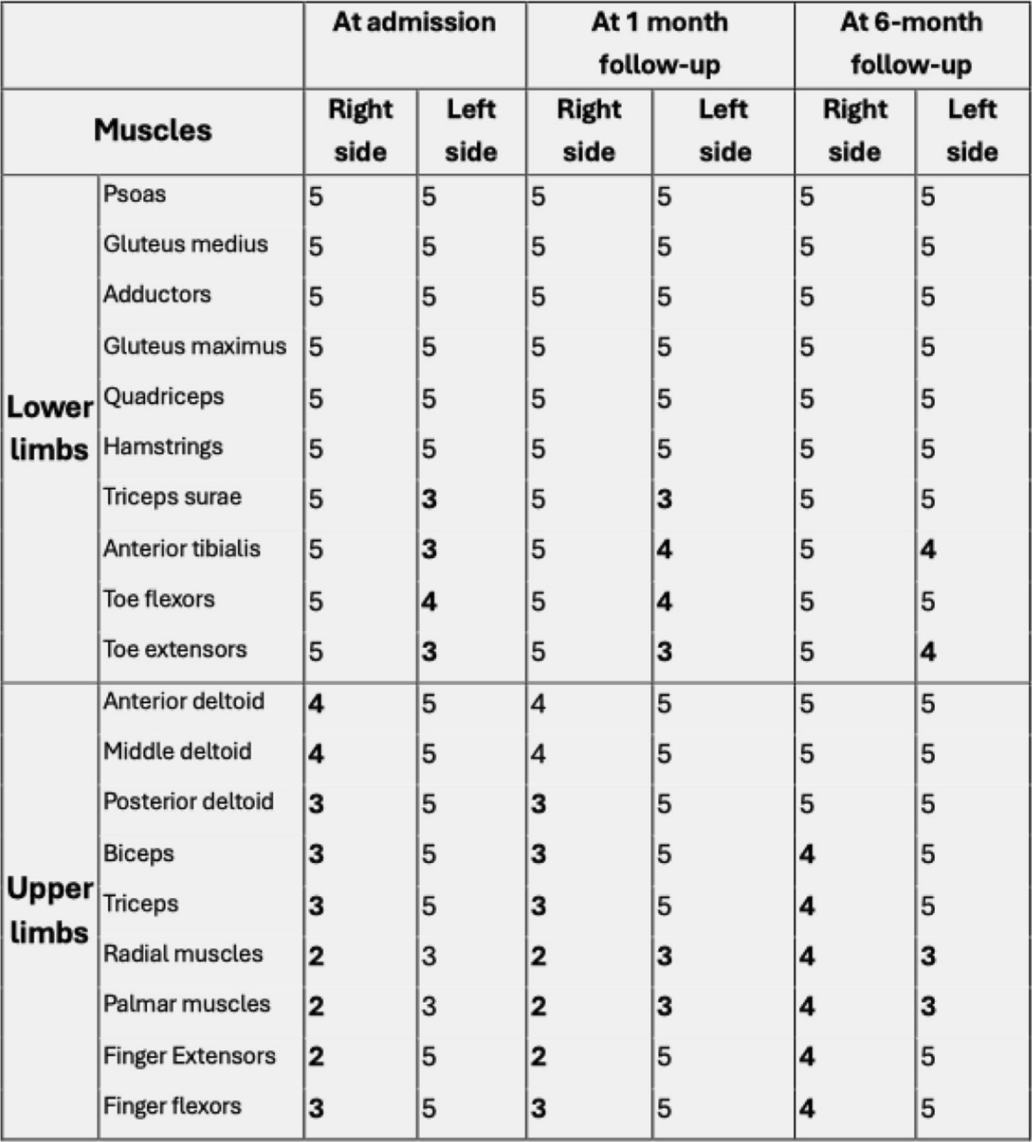
Muscle strength according to the Medical Research Council scale at admission, at 4 weeks and at 6 months, highlighting residual deficits and improvement over time

**Fig. 2:**
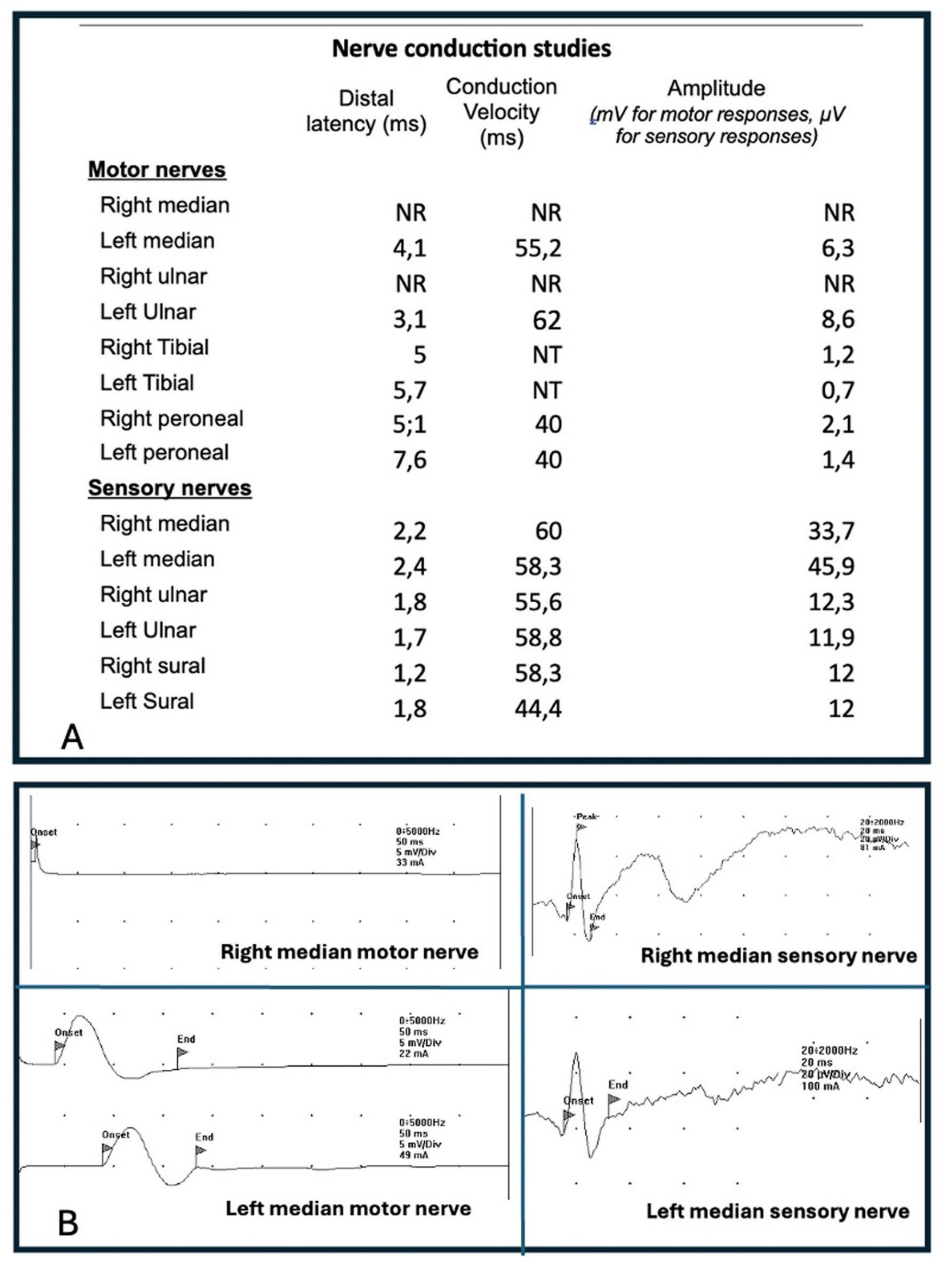
Patient’s nerve conduction study findings. **A** Nerve conduction studies performed in all four limbs, showing pure motor involvement characterized by an asymmetric reduction of compound muscle action potential amplitudes, without abnormalities in conduction velocities or distal latencies. **B** Abolition of the right median motor response, while the left median motor response is preserved, with no abnormalities in the sensory responses. NR, not recorded; NT, not tested

**Fig. 3:**
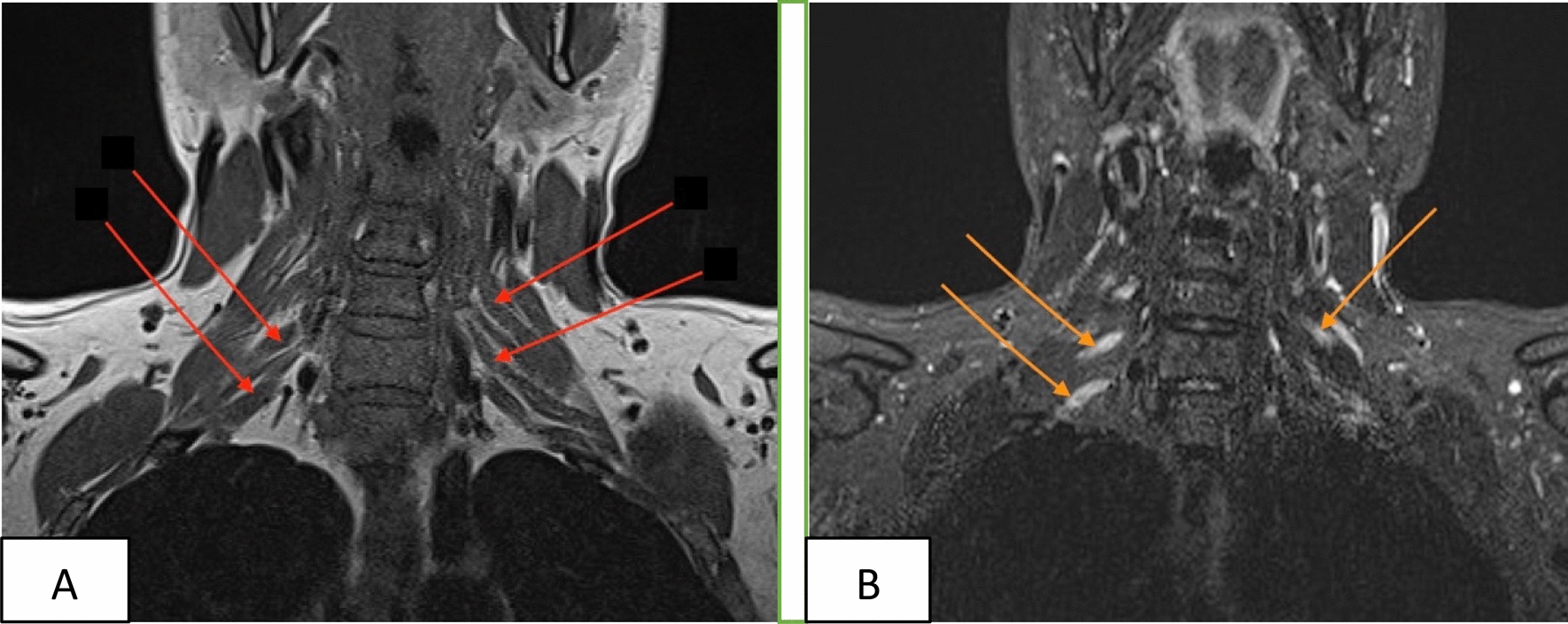
Patient’s brachial plexus magnetic resonance imaging. **A** Bilateral brachial plexus hypertrophy on T1 sequence (red arrows). **B** Bilateral brachial plexus hyperintensity, more marked on the right, on short tau inversion recovery sequences (orange arrows)

Nerve conduction studies demonstrated an asymmetric reduction of compound muscle action potential amplitudes without conduction block or temporal dispersion. Needle electromyography showed chronic neurogenic changes, characterized by large-amplitude motor unit potentials with reduced recruitment in clinically weak and atrophic muscles, while tongue and facial muscles were spared (Fig. 2). Cerebrospinal fluid analysis revealed mild-to-moderate albuminocytologic dissociation with protein level of 0.75 g/L. Serum testing showed positivity for anti-GD3 IgM antibodies. Anti-GM1 antibodies, tested on two separate occasions, yielded equivocal results. Magnetic resonance imaging (MRI) of the brachial plexus, performed after the initiation of the GFD, demonstrated diffuse bilateral hypertrophy on T1-weighted sequences, associated with hyperintensity on short tau inversion recovery (STIR) sequences, without contrast enhancement (Fig. 3). Additional investigations, including serum immunoelectrophoresis and autoimmune screening (antinuclear antibodies, anti-dsDNA antibodies, and IgA anti-transglutaminase antibodies), were negative. Notably, anti-transglutaminase antibodies were assessed while the patient was already on a GFD, which may have contributed to the negative result. Inflammatory markers, vitamin B12 levels, and thyroid function tests were within normal ranges. Viral serologies (HBV, HCV, HIV, and CMV) were negative. A gluten reintroduction followed by duodenal biopsy was proposed to confirm the diagnosis of gluten-related disorder but was declined by the patient owing to fear of relapse of neurological and dermatological symptoms.

In parallel with continued adherence to the GFD, the patient received a single course of intravenous immunoglobulin (IVIg; 0.4 g/kg/day for five consecutive days, without corticosteroids). The neurological assessment using the Neuropathy Impairment Score (NIS) and the Inflammatory Neuropathy Cause and Treatment (INCAT) disability score showed minimal change between baseline and 4 weeks after IVIg administration (NIS: 40.5 to 39; INCAT: 4 to 3), indicating the absence of a significant therapeutic response. Given the strong suspicion of a gluten-related disorder, supported by the presence of dermatitis herpetiformis and chronic diarrhea, as well as the patient’s financial constraints, no further IVIg courses were administered. Although clinical improvement was minimal during the combined IVIg and GFD period, the patient continued to show slow but progressive improvement after IVIg discontinuation while maintaining a strict GFD, suggesting that recovery was primarily attributable to dietary intervention. At the 6-month follow-up and 1 year after the initiation of the GFD, the patient demonstrated continued gradual improvement, confirmed by repeat clinical evaluation (Table 1). On the basis of the overall clinical, electrophysiological, and radiological findings, a diagnosis of MMN secondary to a GRD was considered. No additional treatments were initiated given the patient’s clinical stability and adherence to the GFD; however, regular quarterly clinical and electrophysiological follow-up was planned to monitor for potential disease changes.

## Discussion and conclusion

Gluten ingestion has been associated with a wide spectrum of disorders, collectively referred to as GRD, which have emerged as an increasingly recognized epidemiological entity [4]. The global prevalence of GRD is estimated at approximately 5% of the population and encompasses three main categories: autoimmune (including DH and gluten ataxia), allergic (wheat allergy), and nonceliac gluten sensitivity [4, 5]. DH is considered one of the major extra-intestinal autoimmune manifestations of GRD, supporting a causal link between the neurological manifestations observed in our patient and gluten-related disease. Among peripheral nervous system manifestations, distal symmetric axonal polyneuropathies and small fiber neuropathies are most frequently reported, followed by sensory neuronopathies and multiple mononeuropathies. By contrast, pure motor neuropathy and MMN remain exceptional presentations within the spectrum of GRD [4–6].

The pathophysiology of gluten-related neuropathies, particularly pure motor forms, remains incompletely understood. Several mechanisms have been proposed, including antibody cross-reactivity targeting neuronal antigens, immune complex deposition leading to vascular or tissue injury, direct T cell-mediated cytotoxicity against peripheral nerves, immunotoxic effects of gluten-derived peptides, and nutritional deficiencies secondary to malabsorption that may impair nerve function [7].

Only a limited number of similar cases have been reported to date. In existing literature, MMN-like presentations occurring in the context of gluten-related disorders are generally regarded as autoimmune comorbidities rather than as evidence of a direct causal relationship [8]. Nevertheless, several observations challenge this interpretation. Notably, Rigamonti et al. described two patients with motor neuropathy associated with celiac disease who demonstrated both clinical and electrophysiological improvement following the initiation of a GFD, supporting the possibility of a more direct pathogenic link between gluten exposure and motor neuropathy [2]. Beyond isolated case reports, larger series have also highlighted the occurrence of motor-predominant phenotypes within the spectrum of GRD. In a cohort of approximately 100 patients with gluten neuropathy, nearly 9% exhibited a pure motor neuropathy phenotype, indicating that although uncommon, motor presentations are a recognized manifestation of gluten-related neurological involvement [9]. Furthermore, ALS-like presentations associated with celiac disease have also been described. Lee and colleagues reported a patient initially diagnosed with motor neuron disease whose progressive weakness and fasciculations were ultimately attributed to celiac disease, with subsequent clinical improvement following adherence to a GFD. Recognition of this rare but potentially reversible entity is crucial, as it stands in sharp contrast to alternative diagnoses such as ALS, a condition associated with substantial psychological distress for patients and their families and a profoundly different prognosis, which in several jurisdictions, may even lead to requests for assisted death [10].

Although anti-tissue transglutaminase antibodies are highly sensitive and specific for celiac disease, negative serology does not exclude GRD [11]. In our case, anti-transglutaminase antibodies were negative, and duodenal biopsy could not be performed because the patient declined gluten reintroduction owing to fear of symptom relapse. Possible explanations include seronegative celiac disease or prior dietary gluten restriction. While these factors preclude a definitive diagnosis of celiac disease, the marked and sustained clinical improvement under a GFD strongly supports the diagnosis of a GRD [11].

Clinical improvement in gluten-related neuropathy is most often observed with strict adherence to a gluten-free diet, although the diet does not invariably prevent disease onset nor fully reverse established neurological deficits. A retrospective US study involving 39 patients reported significant neurological improvement under a GFD, and two prospective Italian studies confirmed that adherence was associated with clinical improvement, whereas nonadherence resulted in neurological deterioration [12].

The presence of anti-GD3 antibodies in our patient is consistent with previous reports, as anti-ganglioside antibodies have been detected in up to 65% of neuropathies associated with celiac disease [13, 14]. These antibodies may have mechanistic relevance in motor neuropathy, given their potential to target ganglioside antigens expressed on motor neurons or axons, although their pathogenic role remains uncertain [11]. The persistence of these antibodies after 5 months on a GFD may reflect the slow decline of autoantibody titers, interindividual variability, or assay sensitivity, and does not necessarily indicate ongoing disease activity. Notably, anti-ganglioside antibodies become negative in only approximately half of celiac patients with neurological manifestations following gluten withdrawal.

In our patient, cerebrospinal fluid analysis demonstrated albuminocytologic dissociation, and brachial plexus MRI—performed after initiation of the gluten-free diet—revealed diffuse bilateral hypertrophy on T1-weighted sequences with associated STIR hyperintensity; together, these findings are suggestive of an inflammatory or immune-mediated motor neuropathy. Extensive serological testing excluded other potential autoimmune, nutritional, and infectious causes, supporting a link to a gluten-related etiology. Remarkably, the patient showed significant neurological improvement after starting a GFD, whereas the reintroduction of gluten repeatedly triggered neurological deterioration accompanied by the appearance of dermatitis herpetiformis, a highly characteristic manifestation of gluten-related disorders. This reproducible pattern strongly suggests that gluten exposure was a key driver of the neuropathy rather than a coincidental comorbidity. Moreover, the lack of response to intravenous immunoglobulin therapy, despite inflammatory cerebrospinal fluid changes, further distinguishes this presentation from typical multifocal motor neuropathy. Taken together, these observations indicate that, within the spectrum of gluten-related neurological disorders, MMN may represent a rare but distinct clinical phenotype. Nevertheless, given the limited number of reported cases, this association should be interpreted cautiously, and further studies are warranted to clarify the underlying pathophysiological mechanisms. Screening for GRD may be considered in patients with IVIg-resistant MMN, particularly when supportive clinical features are present. Further studies are required to better define the mechanisms underlying this rare presentation and to establish evidence-based diagnostic and therapeutic strategies.

## Data Availability

All data were accessed through the PubMed search index.

## Abbreviations

MMN: Multifocal motor neuropathy
ESsCD: European Society for the Study of Coeliac Disease
GFD: Free gluten diet
DH: Dermatitis herpetiformis
ALS: Amyotrophic lateral sclerosis
STIR: Short tau inversion recovery
INCAT: Inflammatory Neuropathy Cause and Treatment
NIS: Neuropathy Impairment Score
GRD: Gluten-related disorders
